# Experimental evaluation of absolute quantification in ^99m^Tc‐TRODAT‐1 SPECT/CT brain dopamine transporter (DAT) studies

**DOI:** 10.1002/acm2.13723

**Published:** 2022-07-14

**Authors:** Norasma Amira Zainudin, Nadiah Zulkifli, Khadijah Hamid, Hazlin Hashim, Syahir Mansor

**Affiliations:** ^1^ Departmen of Biomedical Imaging Advanced Medical and Dental Institute Universiti Sains Malaysia Kepala Batas Penang 13200 Malaysia; ^2^ Nuclear Medicine Unit, Advanced Medical and Dental Institute Universiti Sains Malaysia Kepala Batas Penang 13200 Malaysia

**Keywords:** ^99m^Tc‐TRODAT‐1, DAT, quantification, SPECT/CT, SUVr

## Abstract

**Objective:**

To evaluate the quantitative accuracy of clinical brain dopamine transporters (DAT) investigations utilizing ^99m^Tc‐TRODAT‐1 single‐photon emission computed tomography (SPECT)/computed tomography (CT) in experimental and clinical settings.

**Materials and methods:**

The study used an experimental phantom evaluation and a clinical dataset. Three‐dimensional‐ordered subsets expectation–maximization reconstructed the original and resampled datasets using attenuation correction, scatter correction, and resolution recovery. The reconstructed data were analyzed and reported as percentage difference, standardized uptake value reference (SUVr), and a coefficient of variation (CoV). The Taguchi method tested the impact of the three different parameters on signal‐to‐noise ratio (SNR) and SUVr, including number iteration, Poisson resampling, and phantom setup, with and without the plaster of Paris (POP). Six ^99m^Tc‐TRODAT‐1 SPECT/CT scans were acquired in healthy subjects for verification purposes.

**Results:**

The percentage activity difference between the phantom with and without POP is 20% and 5%, respectively. The SUVr reveals a 10% underestimate for both with and without POP. When it comes to the influence of Poisson resampling, the SUVr value for 75% Poisson resampling indicates 10% underestimation on both sides of the caudate and putamen area, with and without POP. When 25% of Poisson resampling is applied, the SUVr value is overestimated (±35%). In the Taguchi analysis, iteration numbers were the most dominant factor with the *F*‐value of 9.41 and the contribution rate of 52.66% (*p* < 0.05) for SNR. In comparison, *F*‐value of 9.1 for Poisson resampled with contribution rate of 58.91% (*p* < 0.05) for SUVr. Reducing counts by 25% from the original dataset resulted in a minimal bias in SUVr, compared to 50% and 75%.

**Conclusion:**

The optimal absolute SPECT/CT quantification of brain DAT studies using ^99m^Tc‐TRODAT‐1 appears achievable with at least 4i10s and SUVr as the surrogate parameter. In clinical investigations, it is possible to reduce the recommended administered dose by up to 25% while maintaining accurate measurement.

## 1 INTRODUCTION

The combination of single‐photon emission computed tomography (SPECT) and computed tomography (CT) has been widely employed in clinical practice and research to diagnose several ailments, including myocardial diseases, endocrine disorders, and central nervous system diseases.^1^ SPECT/CT has been utilized in brain research for the diagnosis and prognosis of cerebral perfusion, receptor investigations, and neurological disorders.^2^, ^3^, ^4^ SPECT, like positron emission tomography (PET), quantifies the radioactivity present in a specific volume of tissue in absolute units, such as kilobecquerels per cubic centimeter.^5^ Photon scatter, photon attenuation, and the collimator detector response are the three fundamental physical limitations that hampered the accuracy of SPECT quantification.^1^, ^2^, ^3^, ^4^, ^5^, ^6^ To reduce error of an absolute activity in SPECT/CT imaging, various compensation methods, such as attenuation correction (AC), scatter correction (SC), and resolution recovery (RR), have been used.^7^

Injection activity, image acquisition, and image reconstruction with specific corrections, such as attenuation and scatter, are the factors that affect SPECT/CT imaging and influence the final reconstructed images.^8^, ^9^, ^10^ The Taguchi method is one of the strategies to optimize the parameter values. This statistical analysis was developed by Dr. Genichi Taguchi and has been used in several research studies to select the optimal parameters from several options.^11^ The Taguchi method can assess the sensitivity and significance of various imaging parameters using a correctly built orthogonal array and the analysis of variance (ANOVA).^12^

Dopamine transporter (DAT) levels were examined with a brain SPECT/CT scan using ^99m^Tc‐TRODAT‐1.^13^ DAT is involved in various brain activities, including learning, motor control, emotion, and executive functions.^2^, ^4^ DAT abnormalities have been linked to various neurological diseases, such as Parkinson's Disease, Huntington's Disease, depression, schizophrenia, and behavioral/chemical addiction.^14^ DAT problems can be diagnosed using a SPECT/CT method that shows a decreased occupancy of a specific radiopharmaceutical in the brain striatum.^15^ Presently, a combination of SPECT/CT and additional separate magnetic resonance imaging (MRI) is used in neurological investigations to improve the anatomical information of the striatum in the brain.^15^, ^16^, ^17^ The anatomical information provided by MRI scans is extremely valuable for visualizing soft tissue features and accurately quantifying radiopharmaceutical uptake.^16^

Moreover, there are numerous ways to reduce the injected activity and the time of the procedure to enhance the patient experience and lessen the anxiety before and during SPECT imaging. The Poisson resampling approach simulates short‐time or low‐dose imaging methods. When modeling count reduction images from full‐count images, Poisson resampling is the approach of choice.^18^ In bone‐and‐lung studies, the Poisson resampling approach has yielded comparable image quality and correct quantitative results for routine SPECT/CT acquisition.^19^ Nonetheless, this approach is currently underutilized in simulating short‐time/lower dose imaging in brain DAT imaging.

This study was conducted to validate the quantitative accuracy through experimental and clinical brain DAT studies using ^99m^Tc‐TRODAT‐1 SPECT/CT.

## 2 MATERIALS AND METHODS

### 2.1 Phantom studies

The modified Jaszczak phantom consists of four spheres, with the largest sphere (*d* = 31.2 mm) representing the caudate anatomical structure and the medium‐sized sphere (*d* = 24.8 mm) representing the putamen anatomical structure. The phantom was covered by 10 mm of plaster of Paris (POP) to simulate the skull and the brain. The phantom setup consists of an equilateral activity concentration ratio between the right and left striatum, which represents the normal radiopharmaceutical uptake in DAT imaging. A total of 68.82 MBq of ^99m^Tc activity was injected into the phantom, which resulted in a final activity concentration of ∼10 kBq/ml. Subsequently, with a final activity concentration of 100 kBq/ml, 10.18 MBq of ^99m^Tc was diluted in 100 ml of saline and filled in the corresponding spheres (left and right caudate and putamen region).

The SPECT/CT images were obtained four times (repeatedly, with and without POP) using GE SPECT/CT NM670 Gamma Camera (GE Healthcare) with and without POP by utilizing a 128 × 128 × 128 matrix with the step‐and‐shoot image acquisition mode. The images were then reconstructed with three‐dimensional ordered subsets expectation–maximization (3D‐OSEM), AC, SC, and RR with iteration numbers of 1, 2, 4, 6, 8, 10, and 20 with a fixed subset of 10. The Poisson resampling was also applied to the original dataset, which yielded counts of 75%, 50%, and 25% of the original data. The images of the reduced counts were likewise reconstructed with 3D‐OSEM using the same corrections, iterations, and subsets as the original counts dataset.

### 2.2 Clinical studies

The brain SPECT/CT studies were performed on six healthy men (mean age, 31.6 year; age range, 22–37 year) who volunteered to participate in the study, for verification purposes. The local ethical committee approved the study, and the subjects provided written informed consent. Both subjects had never smoked and had no neurological diseases or symptoms.

Each patient underwent an MRI procedure utilizing an MRI GE Signa HDx 1.5T (GE Healthcare, Wisconsin, USA) before the SPECT/CT scans to identify the anatomical structure of the caudate and putamen (striatum) region. The gradient echo inversion recovery–isotropic 3D T1 imaging (BRAVO) sequence was used with an image voxel size of 512 × 512 × 64. After the completion of the MRI brain scans, the subjects received a single intravenous injection of ^99m^Tc‐TRODAT‐1 with the activity of 829 ± 47 MBq (mean ± SD). The brain SPECT/CT images were acquired 3–4 h after the injection using the step‐and‐shoot method, 30 s per view, 120 total projection views with a 128 × 128 × 128 image voxel. The images were reconstructed with 3D‐OSEM, AC, SC, and RR and using iteration numbers of 2, 4, 8, 10, and 20 with a fixed subset of 10. Similar to the phantom studies, Poisson resampling was applied to the original dataset, which resulted in 75%, 50%, and 25% counts from the original data. These images were also reconstructed with 3D‐OSEM, and the same reconstruction parameters as the original dataset were used.

### 2.3 Image analysis

The reconstructed SPECT/CT images were analyzed using A Medical Image Data Examiner (version 1.0.4).^20^ For all reconstructed SPECT/CT datasets, four sphere volumes of interest (VOI) with the same diameter as the original sphere were drawn on the right and left of the caudate and putamen region and one in the background for the modified Jaszczak phantom. In phantom studies, the background is considered to be equivalent to the occipital brain region in humans. The placement of VOI in the background was further away from the caudate and putamen regions to model the occipital lobe in the brain. The mean counts for each VOI were acquired and translated to SPECT‐estimated activity concentrations using the conversion factor for this SPECT/CT system.^21^ The percentage of activity difference, SUVr, CoV for each region were calculated using the following formulae:

$$\begin{array}{c} \%\mathrm{Activity}\;\mathrm{difference}=\frac{\mathrm{activity}\;\mathrm{concentration}\;\mathrm{from}\;\mathrm{SPECT}-\mathrm{absolute}\;\mathrm{activity}\;\mathrm{concentration}}{\mathrm{absolute}\;\mathrm{activity}\;\mathrm{concentration}}\times 100 \end{array}$$

$$\mathrm{SUV}=\frac{\mathrm{voxel}\;\mathrm{activity}\;\mathrm{concentration}\times \mathrm{patient}\;\mathrm{weight}}{\mathrm{decay}\;\mathrm{corrected}\;\mathrm{injected}\;\mathrm{activity}}$$

$$\mathrm{SUVr}=\frac{\mathrm{SUV}\;\mathrm{in}\;\mathrm{spher}e_{\left(\mathrm{caudate}\;\mathrm{or}\;\mathrm{putamen}\right)}}{\mathrm{SUV}\;\mathrm{in}\;\mathrm{backgroun}d_{\left(\mathrm{occipital}\right)}}$$

$$\mathrm{CoV}=\frac{\mathrm{standard}\;\mathrm{deviation}\;\mathrm{activity}\;\mathrm{for}\;\mathrm{each}\;\mathrm{region}}{\mathrm{mean}\;\mathrm{activity}\;\mathrm{for}\;\mathrm{each}\;\mathrm{region}}$$

All data were analyzed using the Taguchi method with an orthogonal array design to assess the impact of various factors on the SNR and SUVr. Table 1 lists the three design parameters: iteration number, Poisson resampling %, and phantom setup, with varying degrees of each one. A total of 32 (4 × 4 × 2) combinations of experiments were required. The most suitable orthogonal array, L16 (mixed level), was chosen based on Taguchi's proposal to determine the optimum circumstances and analyze the parameters.^11^, ^12^ Minitab Statistical Software was employed for designing and performing the Taguchi analysis.^22^, ^23^

**TABLE 1:** Three design parameters, including the iteration number, Poisson resampling, and phantom setup, each with different levels

Symbol	Design parameter	Level 1	Level 2	Level 3	Level 4
A	Iteration number	1i10s	2i10s	8i10s	10i10s
B	Poisson resampling	100%	75%	50%	25%
C	Phantom setup	POP	NO POP		

Abbreviation: POP, plaster of Paris.

The SNR for each group of the orthogonal array was calculated using the following formula:

$$\mathrm{SNR}=\frac{\mathrm{mean}\;\mathrm{counts}\;\mathrm{in}\;\mathrm{spher}e_{\left(\mathrm{caudate}\;\mathrm{or}\;\mathrm{putamen}\right)}-\mathrm{mean}\;\mathrm{counts}\;\mathrm{in}\;\mathrm{backgroun}d_{\left(\mathrm{occipital}\right)}}{\mathrm{standard}\;\mathrm{deviation}\;\mathrm{in}\;\mathrm{backgroun}d_{\left(\mathrm{occipital}\right)}}$$

ANOVA was performed to obtain a measure of confidence and to establish which parameters had a statistically significant impact on the final reconstructed images in terms of SNR and SUVr. The effects of the parameters were determined by comparing the *F*‐ratios of each parameter. The greater the *F*‐value, the more dominant the parameter.^12^

The SPECT images were combined with the MRI images in the patient study. The caudate and putamen regions were completely covered by the VOIs, which were manually drawn. One ellipsoid VOI was drawn manually for the occipital region. For each VOI, the SUV_mean_ was obtained, and the SUVr and CoV were calculated. For Poisson resampling of each region, the percentage of activity difference between SUVr and CoV was computed using the following formulae:

$$\%\;\mathrm{difference}\;\mathrm{of}\;\mathrm{SUVr}=\frac{\mathrm{SUVr}\;\mathrm{for}\;\mathrm{each}\;\mathrm{regio}n_{\left(75\%\;\mathrm{or}\;50\%\;\mathrm{or}\;25\%\;\mathrm{resampled}\right)}-\mathrm{SUVr}\;\mathrm{for}\;\mathrm{each}\;\mathrm{regio}n_{\left(\mathrm{original}\right)}}{\mathrm{SUVr}\;\mathrm{for}\;\mathrm{each}\;\mathrm{regio}n_{\left(\mathrm{original}\right)}}\times 100$$

$$\%\;\mathrm{difference}\;\mathrm{of}\;\mathrm{CoV}=\frac{\mathrm{CoV}\;\mathrm{for}\;\mathrm{each}\;\mathrm{regio}n_{\left(75\%\;\text{or}\;50\%\;\text{or}\;25\%\;\text{resampled}\right)}-\mathrm{CoV}\;\mathrm{for}\;\mathrm{each}\;\mathrm{regio}n_{\left(\mathrm{original}\right)}}{\mathrm{CoV}\;\mathrm{for}\;\mathrm{each}\;\mathrm{regio}n_{\left(\mathrm{original}\right)}}\times 100$$

## 3 RESULTS

**FIGURE 1:**
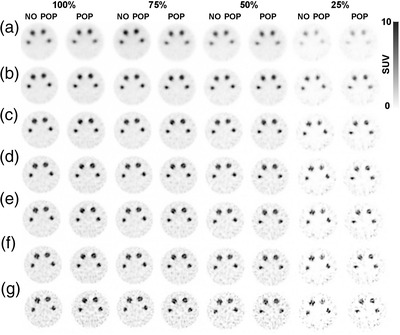
Single‐photon emission computed tomography (SPECT)/computed tomography (CT) images for the original data (100%) with various levels of Poisson resampling (75, 50, and 25%): (a) final reconstructed images with 1i10s, (b) 2i10s, (c) 4i10s, (d) 6i10s, (e) 8i10s, (f) 10i10s, and (g) 20i10s

Figure 1 depicts the transverse SPECT images produced from the modified Jaszczak phantom investigation. The image quality begins to deteriorate after multiple iterations. When the Poisson resampling application is used, the image quality degrades because of decreasing counts and increasing iteration numbers.

### 3.1 Percentage of activity difference

**FIGURE 2:**
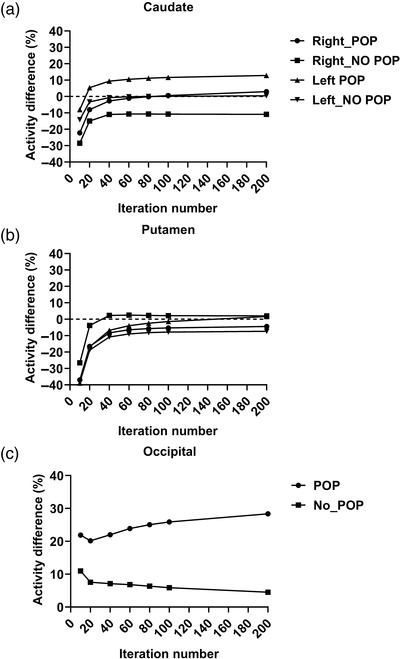
Percentage of activity difference for (a) caudate region, (b) putamen region, and (c) background region for phantom images dataset

**FIGURE 3:**
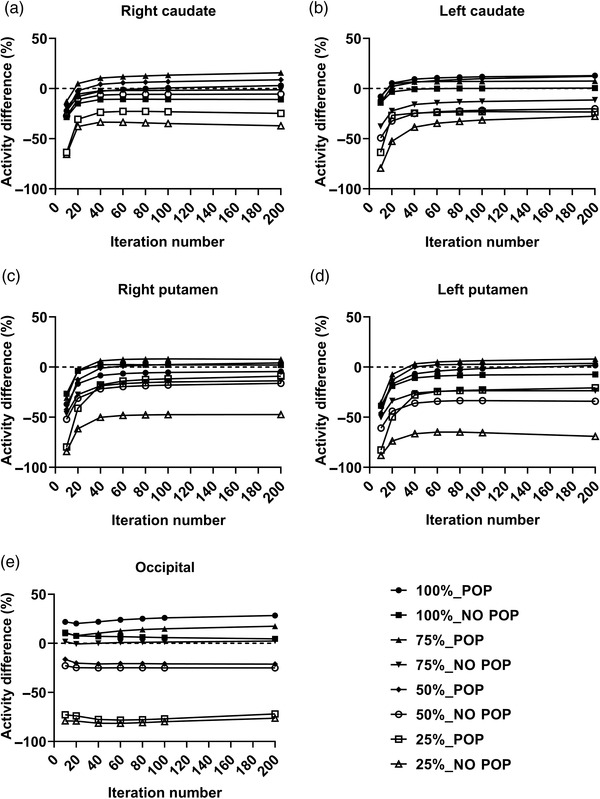
Effect graph for percentage of activity difference in standard data reconstruction and Poisson resampling application for (a) right caudate region, (b) left caudate region, (c) right putamen region, (d) left putamen region, and (e) occipital

Figure 2 depicts the percentage of activity difference between absolute activity concentration and activity concentration predicted from SPECT/CT for each iteration number. According to Figure 2a, the percentage of activity difference between the right and left caudate regions was underestimated by 30% when POP was not used with 10 iterations. However, when POP was used, the percentage of activity difference showed a −30% underestimation for iterations 1 and 2. The percentage of activity difference increased by 20% as the iteration numbers increased to 4, 8, 10, and 20. However, in the putamen region, the percentage of activity difference was in the opposite direction (Figure 2b). When NO POP was used, the percentage of activity difference for the right putamen was overestimated by 2% as the iteration numbers increased to 4, 8, 10, and 20. For iterations 1, 2, 4, 8, 10, and 20, however, the percentage of activity difference suggested a −30% underestimation in the left putamen. From Figure 3, it is suggested that at least 4 iterations and 10 subsets are needed to minimize the bias between absolute and SPECT activity in the original dataset and 75% Poisson resampling dataset, respectively. POP seems to minimally affect the bias in the original dataset and 75% Poisson resampling dataset.

### 3.2 Evaluation of SUVr

**FIGURE 4:**
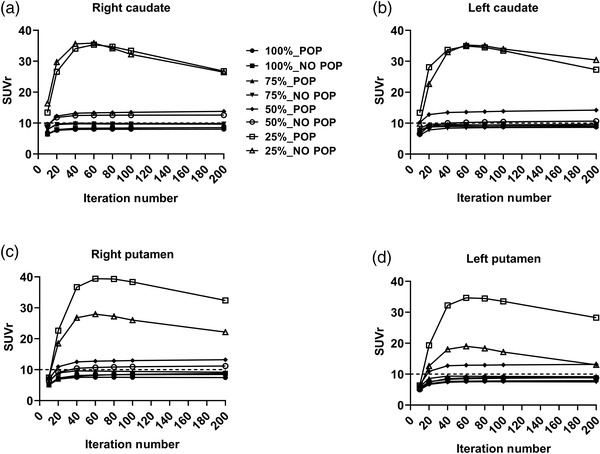
SUVr in original dataset and Poisson resampling dataset for (a) right caudate region, (b) left caudate region, (c) right putamen region, and (d) left putamen region for phantom dataset

According to Figure S1, the SUVr value for each region with and without POP remained underestimated. For the effect of Poisson resampling application, as shown in Figure 4, when including 75% of the original dataset using Poisson resampling, the SUVr value was underestimated by 10% for both sides of the caudate and putamen region with and without POP. When NO POP was used and 50% Poisson resampling was performed, the SUVr value demonstrated an underestimation of within 10% for iterations 1, 2, and 4. However, when the number of iterations increased to 8, 10, and 20, the SUVr values for both the caudate and left putamen were slightly overstated by 50%. Even with 25% Poisson resampling, the SUVr value demonstrated an overestimation of up to 300% (up to four folds; SUVr of 40) for both sides of the caudate and putamen region with and without POP.

### 3.3 Noise characteristic evaluation

**FIGURE 5:**
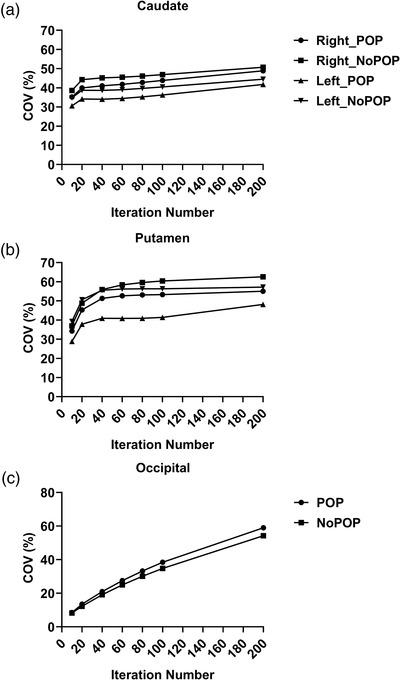
CoV for (a) caudate region, (b) putamen, and (c) occipital for phantom dataset

Figure 5 depicts the background CoV for both sides of the caudate and putamen regions. The CoV value decreased in the caudate and putamen (Figure 5a,b), whereas it increased in the background region (Figure 5c) in phantom with POP as the iteration number increased. However, after four iterations, the CoV values for the right and left sides of the caudate and putamen area were reasonably consistent.

### 3.4 Taguchi method

Table 2 displays the average SNRs and SUVrs for the three levels for iteration number, Poisson resampling, and phantom setup. Among the groups, Group 2 displayed the highest SNR of 92.55. Group 1 presented the lowest SUVr value. Tables 3 and 4 list the main effects for various parameter levels. Higher SNR value and SUVr value closer to the absolute ratio (10) are desirable.

**TABLE 2:** The average of signal‐to‐noise ratios (SNRs) and SUVr over caudate and putamen (31.2‐ and 24.8‐mm spheres) for the 16 groups

Group	Iteration number	Poisson resampling (%)	Phantom setup	SNR	SUVr
1	1i10s	100	NO POP	71.64	5.73
2	1i10s	75	NO POP	92.55	6.38
3	1i10s	50	POP	52.48	7.12
4	1i10s	25	POP	27.74	9.20
5	2i10s	100	NO POP	65.16	7.50
6	2i10s	75	NO POP	75.62	8.23
7	2i10s	50	POP	49.88	10.20
8	2i10s	25	POP	49.54	22.67
9	8i10s	100	POP	25.63	7.72
10	8i10s	75	POP	24.23	9.15
11	8i10s	50	NO POP	27.20	11.49
12	8i10s	25	NO POP	49.72	48.79
13	10i10s	100	POP	22.32	7.78
14	10i10s	75	POP	20.51	9.09
15	10i10s	50	NO POP	23.73	11.56
16	10i10s	25	NO POP	42.38	50.29

Abbreviation: POP, plaster of Paris.

**TABLE 3:** Analysis of variance (ANOVA) results for the three parameters for signal‐to‐noise ratios (SNRs) for the phantom dataset

Source	DF	Adj SS	Adj MS	*F*‐Value	*p*‐Value	Contribution rate (%)
Iteration number	3	3913.9	1304.6	9.43	0.005	52.66
Poisson resampling	3	483.1	161	1.16	0.382	6.50
Phantom setup	1	1928.7	1928.7	13.94	0.006	25.95
Error	8	1107	138.4			
Total	15	7432.6				

*Note*: Degrees of freedom (DF): number of observations in the sample; adjusted sums of squares (Adj SS): measures of variation for different components of the model; adjusted mean squares (Adj MS): measure how much variation a term or a model explains. *F*‐value; determine whether the term is associated with the response. A sufficiently large *F*‐value indicates that the term or model is significant. Contribution rate (%): the percentage that each source in the analysis of variance table contributes to the total sums of squares. Higher percentages indicate that the source accounts for more of the variation in the response. Error: the variability within the groups; total: sum of DF and Adj SS.

**TABLE 4:** Analysis of variance (ANOVA) results for the three parameters for SUVr for the phantom dataset

Source	DF	Adj SS	Adj MS	*F*‐Value	*p*‐Value	Contribution rate (%)
Iteration number	3	439.6	146.53	2.25	0.16	14.54
Poisson resampling	3	1780.8	593.59	9.1	0.006	58.91
Phantom setup	1	280.9	280.88	4.31	0.072	9.29
Error	8	521.6	65.2			
Total	15	3022.9				

*Note*: Degrees of freedom (DF): number of observations in the sample; adjusted sums of squares (Adj SS): measures of variation for different components of the model; adjusted mean squares (Adj MS): measure how much variation a term or a model explains. *F*‐value; determine whether the term is associated with the response. A sufficiently large *F*‐value indicates that the term or model is significant. Contribution rate (%): the percentage that each source in the analysis of variance table contributes to the total sums of squares. Higher percentages indicate that the source accounts for more of the variation in the response. Error: the variability within the groups; total: sum of DF and Adj SS.

#### 3.4.1 ANOVA results

Tables 3 and 4 show the ANOVA results based on SNR and SUVr values for the three parameters. The confidence interval for the ANOVA used in this study was 95%, and the significance level was 0.05. The *F*‐ratios of the different parameters were compared to calculate the influence of the parameters on ANOVA.^12^ According to Table 3, the two significant factors were phantom setup (with and without POP) and iteration number, with the phantom setup having the highest *F*‐value of 13.94 (*p* < 0.05), and the iteration number having an *F*‐value of 9.43 (*p* < 0.05). The Poisson resampling was not statistically significant because the *F*‐value was only 1.16 (*p* > 0.05). Iteration number was the most influential element and accounted for 52.66% of the total contribution rate, followed by phantom setup (25.95%) and Poisson resampling (6.50%).

As indicated in Table 4, the most important factor based on SUVr value was Poisson resampling, which possessed the greatest *F*‐value and contribution rate percentage of 9.1 (*p* > 0.05) and 58.91%, respectively. Iteration number and Poisson resampling were the most beneficial parameters.

### 3.5 Clinical evaluation

**FIGURE 6:**
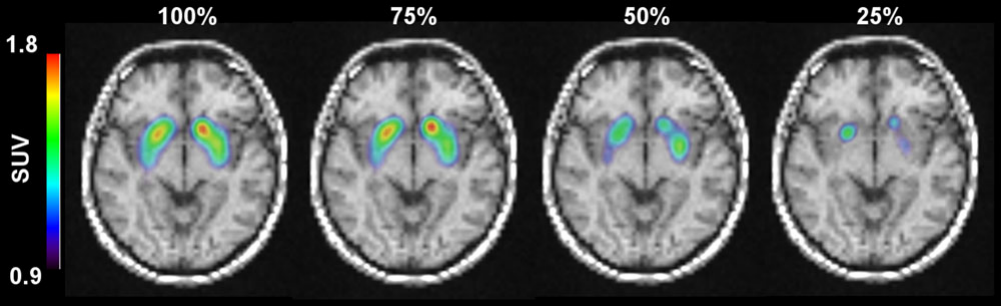
^99m^Tc‐TRODAT‐1 computed tomography (SPECT) overlays on magnetic resonance imaging (MRI) images for various datasets from one of the study subjects. The SPECT images were reconstructed using 4i10s with Butterworth post‐filtering (cutoff value = 0.4 cm^−1^, power value = 10) for better image display.

**FIGURE 7:**
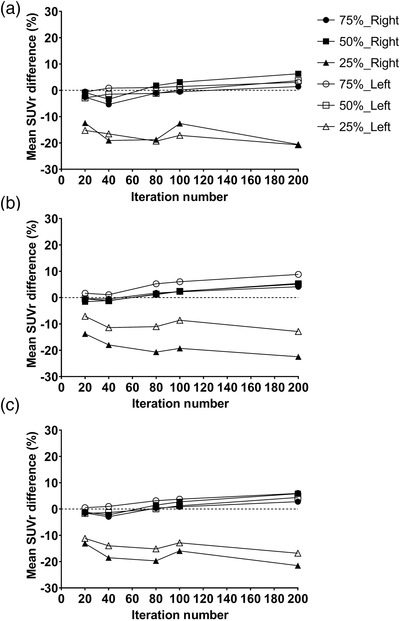
Percentage of mean SUVr difference for different Poisson resampling levels compared with the original dataset for (a) caudate, (b) putamen, and (c) striatum in six normal volunteers

Figure 6 shows a series of fused images for both participants from ^99m^Tc‐TRODAT‐1 SPECT on MRI. The patient research was undertaken to test the effects of iteration number and Poisson resampling based on the findings of the Taguchi analysis. When Poisson resampling was applied to the original dataset, a considerable reduction in the SUVr value was observed, as seen in Figure 7. Resampled data with 75% of the initial counts had a lower level of bias than data with 50% and 25% of the initial counts. The mean difference of SUVr was within 10% of the original dataset when 75% of Poisson resampling was used. The mean difference of SUVr increased by nearly 10% after four iterations. Following 4i10s, the caudate, putamen, and striatum showed a mean differential SUVr fluctuation after 75% and 50% Poisson resampling. When 25% Poisson resampling was used, the mean difference of SUVr in both the caudate and putamen indicated an unstable value. Increasing the number of iterations increased the inaccuracy and amplified the noise (as in CoV), especially in 50% and 25% resampled datasets, as shown in Figure S2.

When 75% Poisson resampling was used, the mean difference for CoV for the background, caudate, and putamen regions remained steady, as shown in Figure S2, even though the iteration numbers increased. When 25% Poisson resampling was used, the rapid variations in CoV reached 170% of the mean difference.

## 4 DISCUSSION

The percentage of activity difference (Figures 2 and 3) for the left putamen and right caudate regions in the experimental section of the study showed an overestimation when POP was used and an underestimation when POP was not employed. This observation could be attributed to the photon AC estimation error when CT is unable to estimate the high‐density structure of POP. The photons released by the radiopharmaceutical interact with the tissues and other materials as they move through the body.^24^, ^25^, ^26^ The degree of photon attenuation depends on the tissue path length and the kind of tissue (e.g., soft tissue, bone, vs. lung) or substance encountered by the photon as it travels from the emission site to the point of detection. Our findings revealed a considerable difference in the effect compared with the previous study, which reported that the use of POP as the brain skull had no meaningful effect.^27^ On the other hand, a study by Benabdallah et al. showed POP was used to mimic the vertebra region for quantification accuracy using various theranostic radionuclides in SPECT/CT phantom studies.^28^ Hence, further experiments with varying POP thicknesses are required. Our findings suggest that when CT erroneously predicts the skull (made of POP), the activity distribution is underestimated. Comparable results were reported when the POP was ignored in the investigation using the PET /magnetic resonance system.^29^ Furthermore, the use of POP increases the noise level in the striatum and background regions when compared with not using POP. The greater the CoV value, the greater the noise in the SPECT images.^17^, ^30^

According to the Taguchi method, iteration number is the most important parameter for qualitative evaluation, whereas Poisson resampling is the most important factor for quantitative evaluation. Two separate techniques (qualitative and quantitative) can be focused on as per the findings of the Taguchi study. Reducing the counts/activity (simulated by Poisson resample) had no statistically significant effect on the outcome when using the SNR as our objective metric; hence, it is more suitable for a qualitative approach. However, the simulation of activity reduction had a statistically significant impact on SUVr as our objective parameter, which reduced the diagnostic value of the brain SPECT/CT studies. The clinical studies was conducted to confirm the findings of the Taguchi analysis. According to the findings, the mean difference for SUVr increased by ±10% as the number of iterations increased. According to the results, the best number of iterations is 4, with a subset of 10. The noise in the SPECT images increased when the count reduction was lowered by up to 75% because of the insufficient number of counts generated from the distribution of radionuclides that interacted with the scintillation detectors.^31^ Hence, a 25% reduction in counts is possible with an acceptable quantitative value and the lowest noise level compared with the original dataset. The dose to a patient's internal organs, such as the liver, spleen, colon, and bladder, increases as the administered dose increases.^32^ Count reduction with reduced given dose or increased imaging time adheres to the notion of “as low as reasonably achievable.” According to this principle, the radiation exposure to the patient can be minimized by reducing the administered dose.

## 5 CONCLUSION

Our findings demonstrate the feasibility of accurately quantifying the ^99m^Tc‐TRODAT‐1 SPECT/CT studies by using SUVr as the surrogate parameter in brain DAT studies. At least 4 iterations with 10 subsets could be used to achieve this result. Furthermore, the counts can be reduced by 25% from the original counts, that is, the administered dose in the clinical studies can be reduced while maintaining accurate quantification relative to the recommended dosage.

## AUTHOR CONTRIBUTION

Conceptualization: Syahir Mansor

Data curation: Norasma Amira Zainudin, Nadiah Zulkifli, and Syahir Mansor

Formal analysis: Norasma Amira Zainudin, Nadiah Zulkifli, and Syahir Mansor

Funding acquisition: Syahir Mansor

Investigation: Norasma Amira Zainudin, Nadiah Zulkifli, Khadijah Hamid, Hazlin Hashim, and Syahir Mansor

Methodology: Norasma Amira Zainudin, Nadiah Zulkifli, Khadijah Hamid, Hazlin Hashim, and Syahir Mansor

Project administration: Norasma Amira Zainudin, Nadiah Zulkifli, and Syahir Mansor

Resources: Norasma Amira Zainudin, Nadiah Zulkifli, and Syahir Mansor

Supervision: Hazlin Hashim and Syahir Mansor

Validation: Norasma Amira Zainudin, Nadiah Zulkifli, Khadijah Hamid, Hazlin Hashim, and Syahir Mansor

Visualization: Norasma Amira Zainudin

Writing original draft: Norasma Amira Zainudin

Writing review and editing: Norasma Amira Zainudin, Nadiah Zulkifli, Khadijah Hamid, Hazlin Hashim, and Syahir Mansor

## ETHICS STATEMENT

This study has been approved by the internal review board (IRB) with an approval number of USM/JEPeM/20020113

## CONFLICT OF INTEREST

No potential conflict of interest relevant to this article was reported.

## Supporting information



Figure S1

Figure S2

## Data Availability

The datasets generated or analyzed during the study are available from the corresponding author on reasonable request.
