# Supplementary figures and images for: Experimental evaluation of absolute quantification in 99mTc‐TRODAT‐1 SPECT/CT brain dopamine transporter (DAT) studies
Source: J Appl Clin Med Phys. 2022 Jul 14;23(8):e13723. doi: 10.1002/acm2.13723 (PMC9359040; doi:10.1002/acm2.13723)

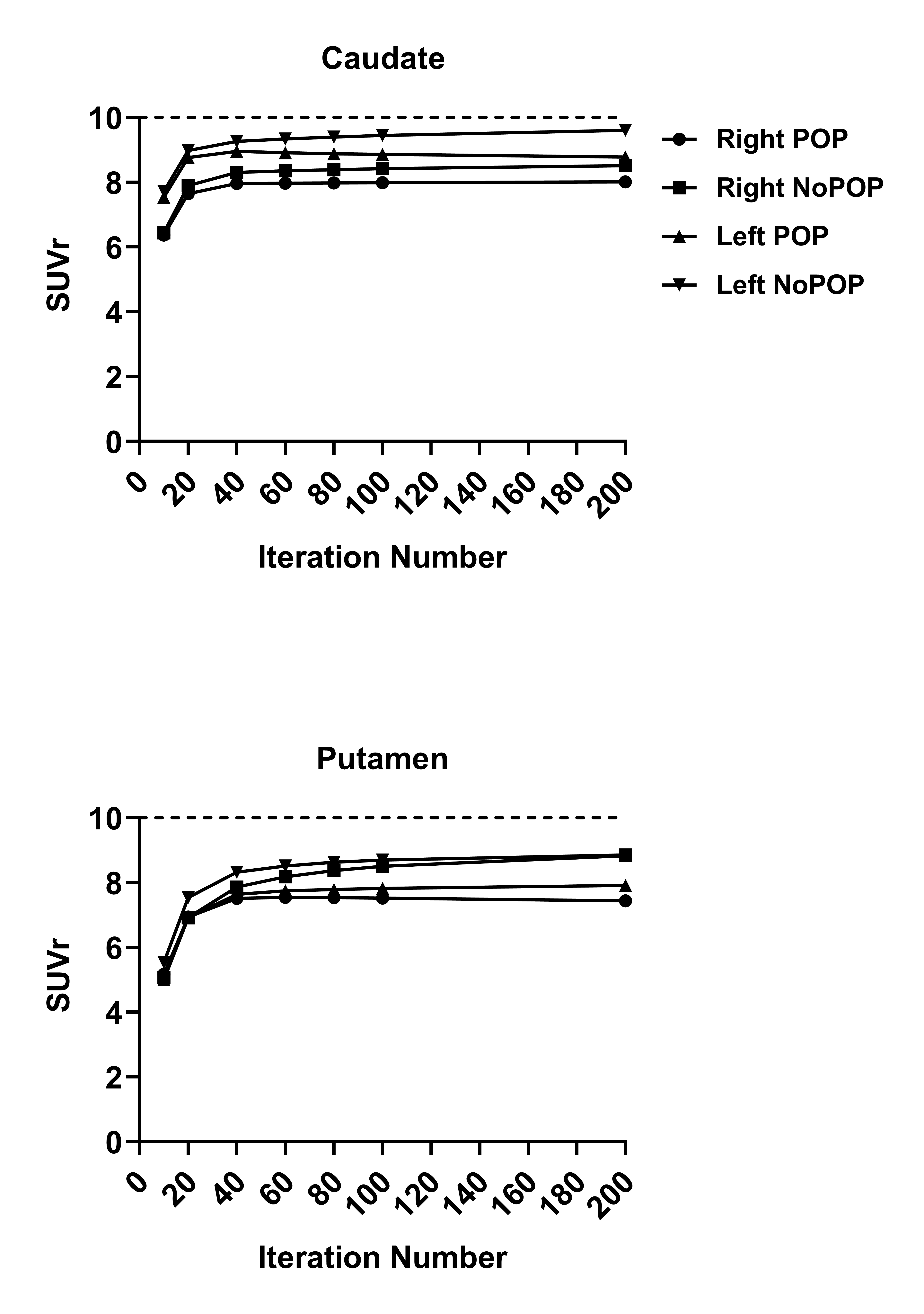

Supplement: Supplementary file 1 — Figure S1 [file ACM2-23-e13723-s002.tif]

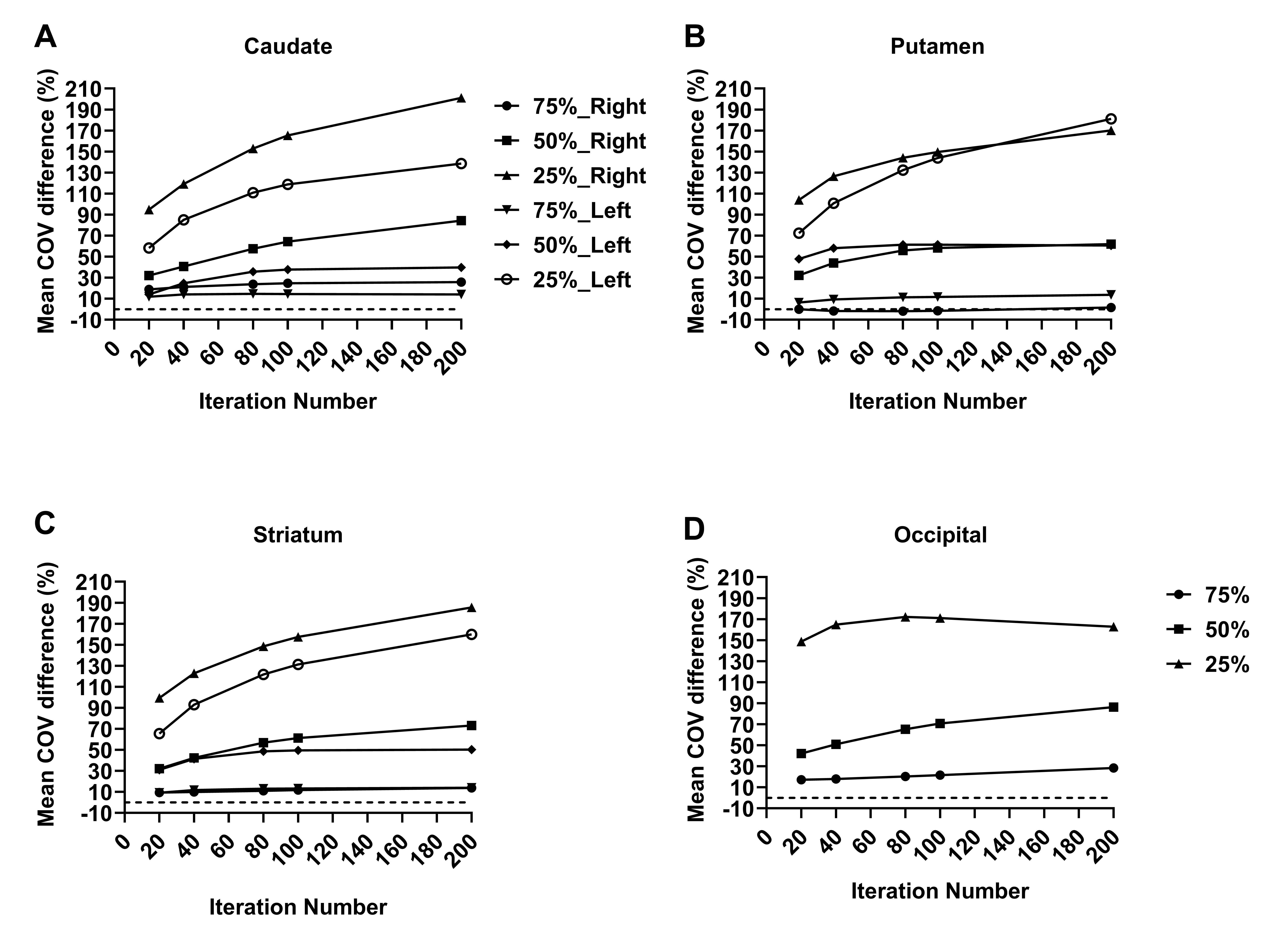

Supplement: Supplementary file 2 — Figure S2 [file ACM2-23-e13723-s001.tif]
